# The quality of preventive care for pre-school aged children in Australian general practice

**DOI:** 10.1186/s12916-019-1455-x

**Published:** 2019-12-06

**Authors:** Louise K. Wiles, Carl de Wet, Chris Dalton, Elisabeth Murphy, Mark F. Harris, Peter D. Hibbert, Charlotte J. Molloy, Gaston Arnolda, Hsuen P. Ting, Jeffrey Braithwaite, on behalf of the CareTrack Kids Investigative Team

**Affiliations:** 1 Australian Institute of Health Innovation, Faculty of Medicine and Health Sciences, Macquarie University, Sydney, NSW Australia; 2 Australian Centre for Precision Health, University of South Australia Cancer Research Institute (UniSA CRI), School of Health Sciences, University of South Australia, Adelaide, SA Australia; 3 South Australian Health and Medical Research Institute (SAHMRI), Adelaide, SA Australia; 4 Healthcare Improvement Unit, Clinical Excellence Division, Queensland Health, Brisbane, QLD Australia; 5 School of Medicine, Griffith University, Gold Coast, QLD Australia; 6 Bupa ANZ, Sydney, NSW Australia; 7 New South Wales Ministry of Health, North Sydney, Sydney, NSW Australia; 8 Centre for Primary Health Care and Equity, Faculty of Medicine, University of New South Wales, Sydney, NSW Australia

**Keywords:** Quality of healthcare, Paediatrics, Preventive medicine, General practice, Process assessment (healthcare), Medical records

## Abstract

**Background:**

Variable and poor care quality are important causes of preventable patient harm. Many patients receive less than recommended care, but the extent of the problem remains largely unknown. The CareTrack Kids (CTK) research programme sought to address this evidence gap by developing a set of indicators to measure the quality of care for common paediatric conditions. In this study, we focus on one clinical area, ‘preventive care’ for pre-school aged children. Our objectives were two-fold: (i) develop and validate preventive care quality indicators and (ii) apply them in general medical practice to measure adherence.

**Methods:**

Clinical experts (*n* = 6) developed indicator questions (IQs) from clinical practice guideline (CPG) recommendations using a multi-stage modified Delphi process, which were pilot tested in general practice. The medical records of Australian children (*n* = 976) from general practices (*n* = 80) in Queensland, New South Wales and South Australia identified as having a consultation for one of 17 CTK conditions of interest were retrospectively reviewed by trained paediatric nurses. Statistical analyses were performed to estimate percentage compliance and its 95% confidence intervals.

**Results:**

IQs (*n* = 43) and eight care ‘bundles’ were developed and validated. Care was delivered in line with the IQs in 43.3% of eligible healthcare encounters (95% CI 30.5–56.7). The bundles of care with the highest compliance were ‘immunisation’ (80.1%, 95% CI 65.7–90.4), ‘anthropometric measurements’ (52.7%, 95% CI 35.6–69.4) and ‘nutrition assessments’ (38.5%, 95% CI 24.3–54.3), and lowest for ‘visual assessment’ (17.9%, 95% CI 8.2–31.9), ‘musculoskeletal examinations’ (24.4%, 95% CI 13.1–39.1) and ‘cardiovascular examinations’ (30.9%, 95% CI 12.3–55.5).

**Conclusions:**

This study is the first known attempt to develop specific preventive care quality indicators and measure their delivery to Australian children in general practice. Our findings that preventive care is not reliably delivered to all Australian children and that there is substantial variation in adherence with the IQs provide a starting point for clinicians, researchers and policy makers when considering how the gap between recommended and actual care may be narrowed. The findings may also help inform the development of specific improvement interventions, incentives and national standards.

## Background

Preventive care is important for optimising every child’s growth and development, especially in the early years [1–5]. While health outcomes for Australian children concord with international studies from the USA [6, 7], the underlying processes and reliability of care are unknown. That is, we do not know whether, or to what extent, evidence- and consensus-based recommended care is actually delivered. Past research suggests that preventive care services afford many benefits to children, including improved healthcare utilisation patterns [8, 9], better parental assessment of child health [8, 9], increased adherence to health-promoting behaviours [9] and enhanced family functioning [8].

In Australia, approximately 5% of all general practitioner (GP) consultations per year are for children aged under 5 years [10]. While a number of different primary care clinicians are available to children and their parents (e.g. child and family health nurses) [11], GPs provide the majority of primary care services for families and children from birth to school entry [12]. This means GPs are ideally placed to play a central role in the early detection of developmental and behavioural problems in children, for disease prevention and health promotion [13].

There is evidence that the care delivered to children is not optimal and varies widely between conditions and healthcare providers. The study by Mangione et al. [7] reported that in the years 1996 to 2000, children received recommended care 68% of the time for acute medical problems, 53% for chronic medical conditions and 41% for preventive healthcare, with an overall average of 47%. Long-term negative sequelae from inadequate preventive screening and care services have been well documented [14–16].

Comprehensive national-level interventions aimed at implementing clinical practice guidelines (CPGs) for preventive child healthcare have produced mixed results; while improvements in general uptake were seen, sustained effects were hampered by issues with adherence to individual core elements, such as making and tracking referrals [17], systematically screening for psychosocial problems and visual disorders [18] and a lack of reliable and feasible metrics [18]. Before future implementation and evaluation of the effect of updated CPGs on clinical practice in Australia, information at a population level regarding whether, and to what extent, recommended healthcare is delivered for children for a range of conditions is needed [6, 19].

The CareTrack Kids (CTK) programme of research sought to address this evidence gap by developing a set of care quality indicators for a range of common paediatric conditions and measuring the frequency with which they were delivered. CTK aimed to assess the quality of care Australian children aged 0–15 years received in 2012 and 2013 by measuring adherence to evidence and consensus-derived recommendations for 17 important common conditions (Table 1) [6].

**Table 1:** Paediatric conditions included in the *CareTrack Kids (CTK)* study (*n* = 17)

Abbreviation	Condition
ABDO	Acute abdominal pain
ADHD	Attention deficit hyperactivity disorder
AGE	Acute gastroenteritis
ANXI	Anxiety
ASTH	Asthma
AUTI	Autism
BRON	Acute bronchiolitis
CROU	Croup
DEPR	Depression
DIAB	Diabetes
ECZE	Eczema
FEVE	Fever
GORD	Gastroesophageal reflux disease
HEAD	Head injury
OTIT	Otitis media
TONS	Tonsillitis
URTI	Upper respiratory tract infection

A separate aim of CTK was to determine the quality of preventive care pre-school aged children received, which is the focus of this study. Our objectives were two-fold: (i) develop and validate preventive care quality indicators and (ii) apply them across GP settings, to measure adherence.

## Methods

The broader methods of the CTK research programme have been described before [6, 19, 20]. This section provides further information specific to this study about preventive care. A more detailed description is provided as a supplementary file (Additional file 1).

### Study design, sample and setting

A retrospective review of a population-based sample of children’s general practice medical records was undertaken. We assessed preventive care delivered in 2012 and 2013 to children aged from 2 months to (and including) 4 years by GPs located in selected health department Health Districts (administrative units through which local health departments deliver health services) across three Australian states (Queensland, New South Wales and South Australia) [6]. Clinical indicators, used to assess the quality of preventive care delivered, were developed by expert panels using a modified Research ANd Development-University of California Los Angeles (RAND-UCLA) consensus-building Delphi process [20, 21].

A sample size for this study was not pre-specified, as preventive care was sampled within medical records that contained occasions of care for one or more of the 17 CTK conditions. Within the selected Health Districts, we advertised the study to GP clinics (making contact with GPs and practice managers) and approached all the providers we could identify through internet searches and via personal contacts. We sampled medical records from the participating general practices for occasions of care for different subsets of eight to nine of the 17 CTK conditions. Further details of our sampling strategy are described in Additional file 1. We estimate that this sampling method covered conditions responsible for approximately 40% of GP consultations with children. Five South Australian GP clinics (located in the same Australian state as the CTK project team members) were recruited for the pilot study. These clinics did not participate in the main study and data were used only for testing and refining methodological processes and the practical application of the indicators; pilot data were not included in the analyses or findings of the main study.

Preventive care was sampled from medical records which were selected because they contained a consultation for one of 17 targeted CTK medical conditions. There are two implications of this approach; firstly, consultations for each condition had different age structures, resulting in different numbers of children born in different periods, ranging from approximately 100 born in 2013 to around 300 in other years. Secondly, the different follow-up periods mean that we sampled proportionately more children at some ages, because these were designated longer follow-up windows (see Additional file 1, section 1.3). Both issues, which manifested as different numbers of children from different age groups being included in the sample (potentially over- or under-representing some), were addressed in analysis by using appropriately calculated weights (see Additional file 1, section 1.5).

### Development and validation of clinical indicators and indicator questions (IQs)

For the purposes of this study, a clinical indicator was defined as ‘a measurable component of a standard or guideline, with explicit criteria for inclusion, exclusion, time frame and practice setting’ [20]. We developed and validated a set of care indicators in a consecutive six-step process [22]:

1. The national and international literature were searched for CPGs about paediatric preventive care [20], and one CPG was found [23].
2. Seventy-one recommendations about specific aspects of care delivery were extracted from the CPG with their associated grades of evidence.
3. The recommendations were reduced to 24 by excluding those that were descriptive statements rather than specific actions; used the terms ‘may’, ‘consider’ and ‘could’; unlikely to be documented in a medical record; or described organisational or systems-based rather than patient-level actions.
4. An expert group of clinicians from the CTK team, consisting of two GPs and one paediatrician, assessed and refined the remaining recommendations during three rounds of the modified RAND-UCLA Delphi process.
5. The draft indicators were assessed and validated by an expert panel of clinicians (two GPs and a paediatrician) who were not part of the CTK team (including definitions for the criteria against which compliance could be measured).
6. The final clinical indicators (*n* = 7) were phrased as specific indicator questions (IQs) (*n* = 43) that could be applied during record review and pilot-tested in general practice.

The expert clinicians (*n* = 6) were recruited through advertisements in medical colleges and professional associations and networks. They assessed the recommendations using a modified RAND-UCLA method for scoring, which is a recognised approach to derive indicators with content, construct and predictive validity in a reliable manner [21]. The experts indicated whether the recommendations and indicators were acceptable (i.e. reflected ‘essential’ Australian clinical practice in 2012–2013); feasible to detect in medical records; and of potential clinical value [20]. In addition to these three criteria, the experts who were not part of the CTK team (step 5), scored indicators on a 9-point Likert scale according to their representativeness of recommended care delivered to children during 2012 and 2013. All experts also recommended for exclusion indicators which duplicated concepts covered in other indicators and provided written feedback (e.g. on the wording of indicators) throughout the process.

### Data collection

Data were collected to assess eligibility for indicator assessment and compliance by eight paediatric nurses (surveyors) who were trained and passed competency evaluations via a 5-day programme. A surveyor manual was developed which included instructions, condition-specific definitions, inclusion and exclusion criteria, and guidance for assessing eligibility of each encounter for relevant indicators. A web-based tool, originally developed for the CareTrack Australia adults study [24, 25] was modified to enter data during the medical record review, which included embedding algorithms to filter indicators by age. As participating general practices were separated by as much as 3000 km (2000 miles), assessing interrater reliability using the actual medical records was not feasible. For the overall CTK analysis, mock medical records were therefore assessed and compared for 6 of the 9 surveyors, and a good level of agreement was found for children’s eligibility for indicator assessment (*K* = 0.76; 95% CI 0.75–0.77; *n* = 1895) and assessment of indicators (*K* = 0.71; 95% CI 0.69–0.73; *n* = 1009). Specifically, for the preventive care mock records, agreement was deemed fair for indicator eligibility (*k* = 0.49; 95% CI 0.41–0.57; *n* = 42) and assessment (*k* = 0.69; 95% CI 0.51–0.86; *n* = 42).

### Analysis

At indicator level, estimates of compliance were measured as the percentage of eligible indicators (i.e. indicators answered either ‘Yes’ or ‘No’) which were scored as ‘Yes’. Compliance results for some clinically related indicators were aggregated as bundles of care. Two different types of bundles were created:

1. *Age group bundles*: At different age ranges, children are recommended to receive different bundles of care. For example, four indicators (PREV36–38 and PREV42) describe specific preventive care interventions recommended for children aged 2 years (e.g. aged at least 2, but less than 3, years). At this age, children should be weighed, have their height measured, have their development and behaviour assessed as specified by the Royal Australian College of General Practitioners [23] and receive the immunisations specified in the schedule prepared by the Australian Department of Health. All four of these indicators would have to be scored ‘Yes’ for the bundle to be scored as being fully compliant with CPG recommendations. When assessing bundle compliance, a survey was only included if there were ‘Yes/No’ responses for all component indicators.
2. *Clinical care bundles*: An alternative way of bundling indicators is according to the clinical care they describe, i.e. assessment or treatment, ignoring the target age. We measured compliance with CPG recommendations relating to anthropometric measurements (assessments) at 2, 4 and 6 months (height/weight/head circumference) and at 12, 18 and 24 months (height and weight). Compliance with CPGs relating to the provision of vaccines (treatment) was measured according to the schedule for patients aged 2, 4, 6, 24 or 48 months. Compliance with the bundles was calculated by counting all the ‘Yes’ responses and dividing them by the total number of ‘Yes’ and ‘No’ responses.

The weighted data were analysed in SAS v9.4, using the SURVEYFREQ procedure. Variance was estimated by Taylor series linearization. State was specified as a stratum, and the primary sampling unit (Health District) was specified as the clustering unit. Exact 95% CIs were generated using the modified Clopper–Pearson method. The overall estimate of recommended preventive care was the weighted average of the individual indicator assessments.

### Ethical considerations

The study was approved by the Royal Australian College of General Practitioners (NREEC 14-008). Australian Human Research Ethics Committees can waive requirements for patient consent for external access to medical records if the study entails minimal risk to healthcare providers and patients [19]; all relevant bodies provided this approval. Participants were protected from litigation by gaining statutory immunity for CTK as a quality assurance activity, from the Federal Minister for Health under Part VC of the Health Insurance Act 1973 (Commonwealth of Australia).

## Results

### Indicators

Our development and validation process resulted in seven clinical indicators, which were formatted into 43 medical record audit IQs. The IQs are listed in Table 3, with additional details in Additional file 2. The 43 IQs were grouped into six age group care bundles (Table 4) and eight clinical care bundles (Table 5).

### Indicator assessments

Of 132,268 possible indicator assessments, 125,561 (94.9%) were automatically filtered out because of age restrictions and an additional 871 (0.7%) were designated as not applicable or ineligible. The surveyors undertook 976 record reviews and 5836 eligible indicator assessments in 80 GP practices. Additional file 3 provides definitions for the criteria against which surveyors marked indicator compliance.

### Characteristics of reviewed records

The individual records of 976 children with one or more preventive care indicators were reviewed (Table 2). Of these, 515 (52.8%) were male. The mean number of indicators was six per record (range 1 to 30).

**Table 2:** Characteristics of the reviewed records

Characteristic	*n* (%)
Gender	
Male	515 (52.8)
Female	461 (47.2)
Eligible IQs per record	
Mean	6
Median	4
Range	1–30

### Quality of preventive care

The assessed quality of treatment for each IQ is shown in Table 3. Quality is reported for all 43 IQs, with compliance ranging from 9.8% for PREV33 (‘Infants aged 18 months had their eyes examined’) to 85.9% for PREV39 (‘Infants aged 2 months received immunisation according to Australian Department of Health and Ageing [DOHA] immunisation schedule’). The interquartile range for quality in the 43 IQs was 25.8 to 61.0%. The overall estimate of the quality of preventive care was 43.3% (95% CI 30.5–56.7).

**Table 3:** Quality of care, by clinical IQ

Indicator ID	IQ description	Number of indicators	Proportion adherent % (95% CI)
PREV01	Infants aged 2 months were weighed.	98	70.3 (55.5, 82.5)
PREV02	Infants aged 4 months were weighed.	91	63.7 (42.3, 81.7)
PREV03	Infants aged 6 months were weighed.	80	65.0 (32.6, 89.5)
PREV04	Infants aged 2 months had their length measured.	99	50.4 (22.3, 78.4)
PREV05	Infants aged 4 months had their length measured.	91	47.5 (19.6, 76.6)
PREV06	Infants aged 6 months had their length measured.	80	62.3 (32.2, 86.7)
PREV07	Infants aged 2 months had their head circumference measured.	99	48.2 (21.1, 76.1)
PREV08	Infants aged 4 months had their head circumference measured.	91	39.0 (19.6, 61.2)
PREV09	Infants aged 6 months had their head circumference measured.	79	61.0 (30.0, 86.5)
PREV10	Infants aged 2 months had their eyes examined.	99	29.8 (18.1, 43.8)
PREV11	Infants aged 4 months had their eyes examined.	91	10.3 (2.8, 24.5)
PREV12	Infants aged 6 months had their eyes examined.	80	22.2 (2.0, 64.1)
PREV13	Infants aged 2 months had their cardiovascular status examined.	99	48.4 (22.1, 75.4)
PREV14	Infants aged 4 months had their cardiovascular status examined.	91	16.2 (5.6, 33.7)
PREV15	Infants aged 6 months had their cardiovascular status examined.	80	27.9 (3.9, 68.7)
PREV16	Infants aged 2 months had their hips, limbs and joints examined.	99	41.2 (31.4, 51.5)
PREV17	Infants aged 4 months had their hips, limbs and joints examined.	91	12.0 (3.3, 28.4)
PREV18	Infants aged 6 months had their hips, limbs and joints examined.	80	19.8 (2.4, 55.6)
PREV19	Infants aged 2 months had their developmental progress examined.	99	40.5 (27.3, 54.8)
PREV20	Infants aged 4 months had their developmental progress examined.	91	22.3 (5.1, 51.6)
PREV21	Infants aged 6 months had their developmental progress examined.	80	28.3 (4.0, 69.2)
PREV22	Infants aged 2 months had any parental concerns documented.	99	39.5 (12.5, 72.4)
PREV23	Infants aged 4 months had any parental concerns documented.	91	32.9 (6.6, 71.1)
PREV24	Infants aged 6 months had any parental concerns documented.	79	37.2 (6.1, 79.7)
PREV25	Infants aged 2 months had their nutrition assessed.	99	48.0 (26.7, 69.8)
PREV26	Infants aged 4 months had their nutrition assessed.	91	42.3 (26.0, 60.0)
PREV27	Infants aged 6 months had their nutrition assessed.	80	24.8 (2.2, 69.4)
PREV28	Infants aged 12 months were weighed.	279	60.3 (43.7, 75.4)
PREV29	Infants aged 18 months were weighed.	140	43.8 (28.3, 60.2)
PREV30	Infants aged 12 months had their height measured.	279	51.2 (34.6, 67.6)
PREV31	Infants aged 18 months had their height measured.	140	25.8 (7.2, 54.5)
PREV32	Infants aged 12 months had their eyes and vision examined.	279	17.6 (13.1, 22.9)
PREV33	Infants aged 18 months had their eyes and vision examined.	141	9.8 (2.7, 23.5)
PREV34	Infants aged 12 months had their developmental progress examined.	279	44.1 (27.1, 62.1)
PREV35	Infants aged 18 months had their developmental progress examined.	141	17.0 (5.2, 37.0)
PREV36	Children aged 2 years were weighed.	289	63.7 (53.8, 72.8)
PREV37	Children aged 2 years had their height measured.	290	39.4 (29.5, 50.0)
PREV38	Children aged 2 years had their development and behaviour assessed.	290	37.4 (27.1, 48.5)
PREV39	Infants aged 2 months received immunisation according to Australian DOHA immunisation schedule.	101	85.9 (66.5, 96.3)
PREV40	Infants aged 4 months received immunisation according to Australian DOHA immunisation schedule.	92	84.6 (65.2, 95.6)
PREV41	Infants aged 6 months received immunisation according to Australian DOHA immunisation schedule.	83	84.4 (69.0, 94.1)
PREV42	Children aged 2 years received immunisation according to Australian DOHA immunisation schedule.	313	72.2 (60.7, 81.9)
PREV43	Children aged 4 years are immunised according to Australian DOHA immunisation schedule.	273	73.5 (56.8, 86.4)
Overall estimate of recommended preventive care		5836	43.3 (30.5, 56.7)

Legend: *ID* identifier, *DOHA* Australian Government Department of Health and Ageing

The estimated quality of recommended care for children at specified age points is summarised in Table 4. Compliance with age-based care bundles for children at 2, 4 and 6 months was 12.4% (95% CI 3.4–28.9), 3.2% (95% CI 0.3–11.9) and 8.7% (95% CI 0.4–36.0), respectively; the component indicator with the lowest compliance was documented eye examination at 2 months (PREV10; 29.8%; 95% CI 18.1–43.8) and 4 months (PREV11; 10.3%; 95% CI 2.8–24.5) and examination of hips, limbs and joints at 6 months (PREV18; 19.8%; 95% CI 2.4–55.6). Compliance with care bundles for children aged 12, 18 and 24 months was 15.4% (95% CI 9.8–22.4), 8.2% (95% CI 2.0–20.7) and 19.5% (95% CI 13.4–26.9), respectively; the component indicator with the lowest compliance was documented eye and vision examination at 12 months (PREV32; 17.6%; 95% CI 13.1–22.9) and 18 months (PREV33; 9.8%; 95% CI 2.7–23.5) and assessment of development and behaviour at 24 months (PREV38; 37.4%; 95% CI 27.1–48.5). Clinical care bundle compliance ranged from 17.9% for visual assessment to 80.1% for immunisation (Table 5). Measured quality of anthropometric measurement care was 52.7% (95% CI 35.6–69.4), with compliance for all other clinical care bundles sitting below 40% (Table 5).

**Table 4:** Quality of care, by age group care bundles

Target age group	Indicator IDs	Number of children	Proportion adherent % (95% CI)
2 months	PREV01, PREV04, PREV07, PREV10, PREV13, PREV16, PREV19, PREV22, PREV25, PREV39	98	12.4 (3.4, 28.9)
4 months	PREV02, PREV05, PREV08, PREV11, PREV14, PREV17, PREV20, PREV23, PREV26, PREV40	90	3.2 (0.3, 11.9)
6 months.	PREV03, PREV06, PREV09, PREV12, PREV15, PREV18, PREV21, PREV24, PREV27, PREV41	78	8.7 (0.4, 36.0)
12 months	PREV28, PREV30, PREV32, PREV34	279	15.4 (9.8, 22.4)
18 months	PREV29, PREV31, PREV33, PREV35	140	8.2 (2.0, 20.7)
24 months	PREV36, PREV37, PREV38, PREV42	288	19.5 (13.4, 26.9)

**Table 5:** Quality of care, by clinical care bundles

Care bundle ID	Clinical care bundle description	Relevant age groups	Indicator IDs	No. of children	No. of indicators	Proportion adherent % (95% CI)
A	Anthropometric measurements (weight, length)	2, 4, 6, 12, 18 and 24 months	PREV01–09, PREV28, 29–31, PREV36–37	679	2225	52.7 (35.6, 69.4)
B	Visual assessment	2, 4, 6, 12 and 18 months	PREV10–12, PREV32–33	389	690	17.9 (8.2, 31.9)
C	Cardiovascular examinations	2, 4 and 6 months	PREV13–15	108	270	30.9 (12.3, 55.5)
D	Musculoskeletal examinations	2, 4 and 6 months	PREV16–18	108	270	24.4 (13.1, 39.1)
E	Developmental progress and behaviour assessments	2, 4, 6, 12, 18 and 24 months	PREV19–21, PREV34–35, PREV38	679	980	31.5 (20.6, 44.0)
F	Consideration of parental concerns	2, 4 and 6 months	PREV22–24	107	269	36.5 (8.7, 73.5)
G	Nutrition assessments	2, 4 and 6 months	PREV25–27	108	270	38.5 (24.3, 54.3)
H	Immunisation	2, 4, 6, 24 and 48 months	PREV39–43	694	862	80.1 (65.7, 90.4)

## Discussion

### Key findings

This is the first known attempt to develop and validate preventive care quality indicators and apply them in general medical practice to measure adherence. We found on average that less than half of all indicator assessments resulted in the provision of recommended preventive care. When assessed as clinical care bundles, estimated GP performance was above this average when providing immunisations and undertaking anthropometric measurements, and below for assessments of nutrition, parental concerns, developmental progress and behaviour, cardiovascular status, the musculoskeletal system and vision. Estimated adherence to CPGs was highest for the complete bundle of preventive care delivered to 24-month-old children and lowest for those aged 4 months.

### Comparative performance

Our findings are broadly consistent with similar studies which assessed recommended preventive care for adults in Australia (42%) [24] and the USA (54.9%) [26]. Despite considerable efforts to promote and facilitate the uptake of CPGs in Australia [2, 6], and the importance of early detection of disease with associated reductions in morbidity and mortality, paediatric preventive care is in line with CPGs less than half the time in both developed and developing health systems [27].

In our study, for the years 2012–2013, recommended preventive care was delivered to Australian children less often than the overall estimate of 59.8% across 17 common medical conditions in the CTK study [6]. It was similar to that reported for preventive care services delivered to American children between 1996 and 2000 (38.3% ‘well-child care’) [7].

Documented compliance with the ‘visual assessment bundle’ was found in less than one in five records. This is the first known measure of paediatric preventive eye care and a potentially concerning finding, given that one in ten Australian children aged 0 to 14 years has a vision disorder [28]. Recommended musculoskeletal examinations also demonstrated low compliance in our CTK population (24.4%; 95% CI 13.1–39.1). While the prevalence of musculoskeletal conditions in children aged less than 4 years of age who present to Australian primary care is estimated at less than 2% [29], given the volume of paediatric consultations in general practice, this nevertheless represents a significant number of children [30]. A complete musculoskeletal assessment has additional benefits as it may predict or detect other conditions in young children early to facilitate prompt initiation of treatment, such as cerebral palsy [31] and torticollis [32].

Documented compliance with recommended developmental progress and behaviour examinations in our study was substantially less than that reported in related literature. For example, assessment of developmental milestones ranged from 49.9% (no electronic medical record or structured well-baby visit record) to 80.3% (use of the Rourke Baby Record (RBR)) [33] for children aged up to 22 months, and 68% in a study where paediatric primary care practices collected their own data [17]. While not directly comparable, the findings suggest lower CTK compliances may be due to differing age distributions of sample populations [33] and clinical contexts, where the imminent commencement of a 9-month intervention to implement American Academy of Pediatrics recommendations for developmental screening and referrals may have raised awareness of the problem [17]. Discrepancies in compliance with recommended nutrition assessments (38.5% in CTK, 69.8% in [33]) may reflect disparate sample age groups.

Documented compliance was highest with the ‘immunisation’ bundle, with approximately four in five children receiving the recommended care. This is higher than rates reported in two studies from the USA that used comparable data collection methods. In a 2007 study [7], only 49.8% of children were fully immunised, but CTK did not follow infants longitudinally. In a 2015 study [34], it was reported that 78.4% of newborns and 56% of infants and toddlers were up-to-date with their immunisation status. On the other hand, higher rates of immunisation coverage have been reported in other primary care settings, including New Zealand (95.8%) [35] and Belgium (96.6–99.5%) [36]. The variation in immunisation rates between studies might be because of sampling considerations (i.e. a single-site vs the CTK population-based survey) [35], different data sources (e.g. electronic vaccine ordering and registration system vs CTK medical record audits) [36], specific vaccinations [36] and true inter-country differences [34, 36]. Another consideration may be that our CTK study did not formally evaluate the timeliness of vaccination [35, 36]; while a range of age groups (ages 2, 4, and 6 months and 2 and 4 years) were covered by our set of IQs, we did not specifically gather data on compliance with immunisation schedules for children between the ages of 6 months and 2 years, or those aged 3 years. This means that if CTK children had experienced delays in their immunisation but vaccines were complete by the ages of 2 or 4 years, our IQs still considered vaccinations to have been delivered on schedule [37].

### Implications and next steps

Barriers to the provision of well-child healthcare have been proffered by Australian GPs and include time constraints, the financial status of families in lower socioeconomic groups who may not be able to cover gap payments, a lack of knowledge around the availability of and access to paediatric services, and fragmented care as a result of poor interflow of information among healthcare providers [2]. Shortfalls in the financial recompense available to GPs who provide preventive paediatric or ‘well-child’ care may be an additional barrier [38]; the *Healthy Kids Check* [39] which was operational during the study period enabled a national government rebate (Medicare) to be claimed by GPs only when a child was 4 years of age and had an up-to-date patient history, a complete immunisation schedule and been given a health promotion booklet [40]. However, the Check was retired in 2015 due to underperformance, cost blowouts and duplication of state and territory-based programs [41]. There are many potential interventions and initiatives to address barriers and improve preventive care [42], but success is variable, context-dependent and, too often, not rigorously assessed [43–49]. It is unlikely that any single intervention aimed at improving quality of care will yield significant or sustained benefits, and there is a need to engage, empower and support parents of children with knowledge of the importance, and availability of, resources for preventive health in early childhood to help to optimise preventive care for young children [2, 42, 50, 51]. Two of the key requirements for future interventions are therefore standards and reliable metrics—both components are potentially provided by this study.

Lack of agreed definitions and established clinical standards of what constitutes recommended (or quality) preventive care is a barrier to best practice. Internationally agreed standards on well-child management and preventive care would allow comparison between healthcare systems and facilitate evaluation of the effectiveness of interventions. The Royal Australian College of General Practitioners (RACGP) have produced a national CPG for preventive activities in children and young people [52]. The development and implementation of guideline recommendations as measurable standards is strongly advocated as a means by which to initially audit and then ultimately improve quality of care [2, 24, 53].

A way forward could be the use of integrated e-health medical records embedded with clinical standards to help facilitate the provision of recommended care, consistency and completeness of documentation and enable large-scale surveillance or regionally based audits of current practice as well as for responsiveness to national initiatives to improve healthcare [24]. Future initiatives and interventions must target practitioners and consumers (paediatric patients and their parents) to optimise individual patient outcomes in addition to process measures.

### Strengths and limitations

The key strength of the CTK study is that it was designed to be representative of a broad segment of the Australian population rather than a convenience or purposive sample. However, we did not collect sociodemographic data or randomly select all medical records to be reviewed; records were only sampled if they were identified as having a consultation for one of 17 common paediatric conditions covering approximately 40% of GP consultations with children. This sampling method could bias our estimates, if the sampled conditions are correlated with the quality of preventive care. Therefore, our results are only strictly generalisable to children who have one or more of the 17 conditions sampled in CTK at some point in the 2-year period of interest. This approach was used, rather than selecting another random sample of children without one of the 17 CTK conditions, to minimise the workload on administrative staff at participating general practices who were tasked with generating de-identified lists of eligible children and facilitating surveyor access of their records.

We included only GP records and occasions of service (including care provided by general practice nurses). Our findings therefore do not reflect the quality of preventive care delivered by other types of primary care clinicians. For example, children receiving immunisations from community child-health clinic nurses were not captured in this study as their treatment may not have been documented in general practice records [54]. In Australia, GPs are the predominant provider of paediatric vaccination services delivering nearly three-quarters of all vaccinations for children aged 0–6 years, which is in addition to being regularly contracted by State Governments to deliver the childhood vaccination programs provided through schools and community centres [55]. An investigation of the quality of preventive care provided to older children would be an important focus of future research as no other primary care clinicians provide well-child care for this age group. Integrated e-health medical records which record both GP and ‘maternal and child health’ data would facilitate the feasibility of such an endeavour.

The IQs were drawn from one CPG relevant to Australian general practice in 2012–2013, which limits the applicability of the IQs to other contexts and settings. Six clinical experts (three within the CTK team and three external to it) participated in the Delphi process to develop the IQs. This may have adversely affected the face validity of ‘preventive care’ IQs. The IQs, which were developed and validated using rigorous methods to measure compliance, measure most, but not all, aspects of preventive care for children aged from 2 months to 4 years. Age group bundles will have lower compliance scores than clinical care bundles because the former are linked by ‘AND’ statements; compliance with an age group bundle cannot be higher than the compliance for the IQ with the lowest compliance. Clinical care bundles, on the other hand, are weighted averages of all the component indicators assessed. The use of bundles as composite measures in addition to individual IQs provide an alternative perspective on care quality [56].

Our findings provide a snapshot in time, and compliance rates may have changed since 2013. Despite considerable investment in developing and disseminating comprehensive clinical practice guidelines [52], the latest Australian Research Alliance for Children and Youth (ARACY) report card suggests that some outcomes (e.g. immunisation against measles and whooping cough) have worsened since the last report in 2013 [57]. The CTK study has relied on process (rather than outcome-based) indicators to audit care as documented within the medical record. Previous research has suggested that medical records may underestimate the quality of care provided by healthcare services, with doctors being more likely to document some aspects of care (e.g. medication, immunisations) than others in medical records (e.g. patient history information provided to patients) [58, 59]. The record review method depends on the quality of clinical records and surveyor characteristics; however, we sought to pre-emptively address this by using a two-stage structured record review method, training and testing surveyors prior to data collection and providing a coding manual.

## Conclusion

Our findings that preventive care is not reliably delivered to all Australian children and that there is substantial variation in adherence with the indicators provide a starting point for clinicians, researchers and policy makers when they consider how the gap between recommended and actual care may feasibly be narrowed. The findings may also help inform the development of specific improvement interventions, incentives and national standards.

## Supplementary information

**Additional file 1.** Additional details relating to study methods. 1.1 Sample size. 1.2 Sampling Process. 1.3 Sampling of records. eTable 1.1. Number of indicators and follow-up duration for children of different ages. 1.4 Sampling weights. 1.5 Analysis. 1.6 Results. eTable 1.2. Sample size for each indicator by target age for assessment/intervention. References.

**Additional file 2.** Listing of indicator characteristics. eTable 2.1. Characteristics, by clinical indicator, 2012–2013.

**Additional file 3.** Definitions for the criteria against which reviewers marked indicator compliance.

## Data Availability

Patient data in this study are not publicly available as they were collected from medical records examined by the research team without seeking individual consent. Four ethics committees approved this data extraction without consent and would need to approve the release of data collected by the project, to ensure protection of both healthcare providers and individual patients. Most of the data used for calculation of weights is owned by third parties, and its release will be subject to third party approvals from three state health departments (populations by health district, total ED presentations and inpatient admission numbers by hospital, percentage of ED admissions by condition), the Australian Government Department of Human Services (total number of consultations with children by general practitioners and community paediatricians), the Australian Paediatric Research Network (percentage of consultations for each condition by community paediatricians) and the Bettering the Evaluation and Care of Health programme (percentage of consultations by condition for general practice). Data will be made available by the authors upon reasonable request, and with the approval of all bodies from whom permissions are required.

## References

[1] Doyle O, Harmon CP, Heckman JJ, Tremblay RE (2009). Investing in early human development: timing and economic efficiency. Econ Hum Biol.

[2] Jeyendra A, Rajadurai J, Chanmugam J, Trieu A, Nair S, Baskaran R (2013). Australian general practitioners' perspectives on their role in well-child health care. BMC Fam Pract.

[3] Moore T, Skinner A (2016). An integrated approach to early childhood development.

[4] Tylee A, Haller DM, Graham T, Churchill R, Sanci LA (2007). Youth-friendly primary-care services: how are we doing and what more needs to be done?. Lancet..

[5] World Health Organization, United Nations Children's Fund (UNICEF) (2012). Early childhood development and disability: a discussion paper.

[6] Braithwaite J, Hibbert PD, Jaffe A, White L, Cowell CT, Harris MF (2018). Quality of health care for children in Australia, 2012-2013. JAMA..

[7] Mangione-Smith R, DeCristofaro AH, Setodji CM, Keesey J, Klein DJ, Adams JL (2007). The quality of ambulatory care delivered to children in the United States. N Engl J Med.

[8] Kuhlthau KA, Bloom S, Van Cleave J, Knapp AA, Romm D, Klatka K (2011). Evidence for family-centered care for children with special health care needs: a systematic review. Acad Pediatr.

[9] Hadland SE, Long WE (2014). A systematic review of the medical home for children without special health care needs. Matern Child Health J.

[10] Britt H, Miller GC, Valenti L, Henderson J, Bayram C, Gordon J (2016). The changing face of Australian general practice across the decades. Aust Fam Physician.

[11] Schmied V, Mills A, Kruske S, Kemp L, Fowler C, Homer C (2010). The nature and impact of collaboration and integrated service delivery for pregnant women, children and families. J Clin Nurs.

[12] Goldfeld S, Wright M, Oberklaid F (2003). Parents, infants and health care: utilization of health services in the first 12 months of life. J Paediatr Child Health.

[13] Oberklaid F, Efron D (2005). Developmental delay-identification and management. Aust Fam Physician.

[14] Garner AS, Shonkoff JP (2012). Early childhood adversity, toxic stress, and the role of the pediatrician: translating developmental science into lifelong health. Pediatr..

[15] Graungaard AH, Skov L (2007). Why do we need a diagnosis? A qualitative study of parents' experiences, coping and needs, when the newborn child is severely disabled. Child Care Health Dev.

[16] Riou EM, Ghosh S, Francoeur E, Shevell MI (2009). Global developmental delay and its relationship to cognitive skills. Dev Med Child Neurol.

[17] King TM, Tandon SD, Macias MM, Healy JA, Duncan PM, Swigonski NL (2010). Implementing developmental screening and referrals: lessons learned from a national project. Pediatr..

[18] Fleuren MAH, van Dommelen P, Dunnink T (2015). A systematic approach to implementing and evaluating clinical guidelines: the results of fifteen years of Preventive Child Health Care guidelines in the Netherlands. Soc Sci Med.

[19] Hooper TD, Hibbert PD, Mealing N, Wiles LK, Jaffe A, White L (2015). CareTrack Kids—part 2. Assessing the appropriateness of the healthcare delivered to Australian children: study protocol for a retrospective medical record review. BMJ Open.

[20] Wiles LK, Hooper TD, Hibbert PD, White L, Mealing N, Jaffe A (2015). CareTrack Kids—part 1. Assessing the appropriateness of healthcare delivered to Australian children: study protocol for clinical indicator development. BMJ Open.

[21] Fitch K, Bernstein SJ, Aguilar MD, Burnand B, LaCalle JR (2001). The RAND/UCLA appropriateness method user's manual.

[22] Wiles LK, Hooper TD, Hibbert PD, Molloy C, White L, Jaffe A (2019). Clinical indicators for common paediatric conditions: processes, provenance and products of the CareTrack Kids study. PLoS One.

[23] Royal Australian College of General Practitioners (2013). Guidelines for preventive activities in general practice 8th edition - preventive activities in children and young people.

[24] Runciman WB, Coiera EW, Day RO, Hannaford NA, Hibbert PD, Hunt TD, Westbrook JI, Braithwaite J (2012). Towards the delivery of appropriate health care in Australia. Med J Aust.

[25] Hunt TD, Ramanathan SA, Hannaford NA, Hibbert PD, Braithwaite J, Coiera E (2012). CareTrack Australia: assessing the appropriateness of adult healthcare: protocol for a retrospective medical record review. BMJ Open.

[26] McGlynn EA, Asch SM, Adams J, Keesey J, Hicks J, DeCristofaro A (2003). The quality of health care delivered to adults in the United States. N Engl J Med.

[27] Liu L, Oza S, Hogan D, Perin J, Rudan I, Lawn JE (2015). Global, regional, and national causes of child mortality in 2000-13, with projections to inform post-2015 priorities: an updated systematic analysis. Lancet..

[28] Australian Institute of Health and Welfare (2016). Australia’s health 2016. Australia’s health series no. 15.

[29] Henschke N, Harrison C, McKay D, Broderick C, Latimer J, Britt H (2014). Musculoskeletal conditions in children and adolescents managed in Australian primary care. BMC Musculoskelet Disord.

[30] Britt H, Miller G, Henderson J, Bayram C, Valenti L, Harrison C (2013). General practice activity in Australia 2012–13. General practice series no.33.

[31] Bosanquet M, Copeland L, Ware R, Boyd R (2013). A systematic review of tests to predict cerebral palsy in young children. Dev Med Child Neurol.

[32] Stellwagen L, Hubbard E, Chambers C, Jones KL (2008). Torticollis, facial asymmetry and plagiocephaly in normal newborns. Arch Dis Child.

[33] Rourke L, Godwin M, Rourke J, Pearce S, Bean J (2009). The Rourke Baby Record Infant/Child Maintenance Guide: do doctors use it, do they find it useful, and does using it improve their well-baby visit records?. BMC Fam Pract.

[34] Pati S, Ladowski KL, Wong AT, Huang J, Yang J (2015). An enriched medical home intervention using community health workers improves adherence to immunization schedules. Vaccine..

[35] Reynolds G, Timo M, Dev A, Poole T, Turner N (2014). Effective general practice: audit and feedback for the primary series of immunisations. J Prim Health Care.

[36] Lernout T, Theeten H, Hens N, Braeckman T, Roelants M, Hoppenbrouwers K, Van Damme P (2014). Timeliness of infant vaccination and factors related with delay in Flanders, Belgium. Vaccine.

[37] Hughes MM, Katz J, Englund JA, Khatry SK, Shrestha L, LeClerq SC, Steinhoff M, Tielsch JM (2016). Infant vaccination timing: beyond traditional coverage metrics for maximizing impact of vaccine programs, an example from southern Nepal. Vaccine..

[38] Alexander KE, Brijnath B, Mazza D (2014). Barriers and enablers to delivery of the Healthy Kids Check: an analysis informed by the Theoretical Domains Framework and COM-B model. Implement Sci.

[39] Australian Government: Department of Health. Medicare Benefits Schedule (MBS): Healthy Kids Check. Available at: www1.health.gov.au/internet/main/publishing.nsf/Content/99F39989B0B9D694CA257BF000209AE2/$File/HKC%20Checklist%20-%2030%20April%202014.pdf. (2014).

[40] Alexander K, Mazza D (2010). How to perform a 'Healthy kids Check'. Aust J Gen Pract.

[41] Alexander KE, Mazza D (2015). Scrapping the healthy kids check: a lost opportunity. Med J Aust.

[42] Williams N, Woodward H, Majeed A, Saxena S (2011). Primary care strategies to improve childhood immunisation uptake in developed countries: systematic review. JRSM Short Reports.

[43] Eijkenaar F, Emmert M, Scheppach M, Schoffski O (2013). Effects of pay for performance in health care: a systematic review of systematic reviews. Health Policy.

[44] Flodgren G, Eccles MP, Shepperd S, Scott A, Parmelli E, Beyer FR. An overview of reviews evaluating the effectiveness of financial incentives in changing healthcare professional behaviours and patient outcomes. Cochrane Database Syst Rev. 2011. 10.1002/14651858.cd009255:Cd009255. 10.1002/14651858.CD009255 PMC4204491 21735443

[45] Herzog R, Alvarez-Pasquin MJ, Diaz C, Del Barrio JL, Estrada JM, Gil A (2013). Are healthcare workers' intentions to vaccinate related to their knowledge, beliefs and attitudes? A systematic review. BMC Public Health.

[46] Ivers N, Jamtvedt G, Flottorp S, Young JM, Odgaard-Jensen J, French SD, et al. Audit and feedback: effects on professional practice and healthcare outcomes. Cochrane Database Syst Rev. 2012;6. 10.1002/14651858.CD000259.pub3 PMC11338587 22696318

[47] Steel N, Willems S (2010). Research learning from the UK Quality and Outcomes Framework: a review of existing research. Qual Prim Care.

[48] Van Herck P, De Smedt D, Annemans L, Remmen R, Rosenthal MB, Sermeus W (2010). Systematic review: effects, design choices, and context of pay-for-performance in health care. BMC Health Serv Res.

[49] Ward K, Hull BP, Leask J (2013). Financial incentives for childhood immunisation - a unique but changing Australian initiative. Med J Aust.

[50] Alexander KE, Brijnath B, Mazza D (2015). Parents’ decision making and access to preventive healthcare for young children: applying Andersen's model. Health Expect.

[51] Wakefield MA, Loken B, Hornik RC (2010). Use of mass media campaigns to change health behaviour. Lancet..

[52] Royal Australian College of General Practitioners (2016). Guidelines for preventive activities in general practice.

[53] Melnyk BM, Grossman DC, Chou R, Mabry-Hernandez I, Nicholson W, DeWitt TG (2012). USPSTF perspective on evidence-based preventive recommendations for children. Pediatr..

[54] Kearney L, Fulbrook P (2012). Open-access community child health clinics: the everyday experience of parents and child health nurses. J Child Health Care.

[55] Australian Medical Association (AMA). Position statement on vaccinations outside of general practice. Available at: https://ama.com.au/position-statement/vaccinations-outside-general-practice-2016. (2016). Accessed 11 Sept 2019.

[56] de Wet C, McKay J, Bowie P (2012). Combining QOF data with the care bundle approach may provide a more meaningful measure of quality in general practice. BMC Health Serv Res.

[57] Australian Research Alliance for Children and Youth. Report Card: The wellbeing of young Australians 2018. Available at https://www.aracy.org.au/publications-resources/command/download_file/id/361/filename/ARACY_Report_Card_2018.pdf. (2018). Accessed 20 Mar 2018.

[58] Owen J, Conway R, Silke B, O’Riordan D (2015). Medical record documentation among interns: a prospective quality improvement study. Ir Med J.

[59] Soto CM, Kleinman KP, Simon SR (2002). Quality and correlates of medical record documentation in the ambulatory care setting. BMC Health Serv Res.

