# Supplementary material for: The quality of preventive care for pre-school aged children in Australian general practice
Source: BMC Med. 2019 Dec 6;17:218. doi: 10.1186/s12916-019-1455-x (PMC6896286; doi:10.1186/s12916-019-1455-x)
Supplement: Supplementary file 1 — Additional file 1. Additional details relating to study methods. 1.1 Sample size. 1.2 Sampling Process. 1.3 Sampling of records. eTable 1.1. Number of indicators and follow-up duration for children of different ages. 1.4 Sampling weights. 1.5 Analysis. 1.6 Results. eTable 1.2. Sample size for each indicator by target age for assessment/intervention. References. [file 12916_2019_1455_MOESM1_ESM.docx]

**Additional file 1: Additional details relating to study methods**

The main CTK paper^[1]^ and its associated online appendix detail the methods of the larger study, which generated the data reported in this paper. Selected methods specifically relevant to preventive care are described below.

## 1.1 Sample size

CTK targeted 6400 medical records for 17 important clinical conditions in children across four types of healthcare setting: General Practitioners (GPs) and specialist paediatricians in the community and, in hospitals, emergency department presentations and inpatient admissions. Four hundred records were targeted for each of 15 clinical conditions (*Acute abdominal pain, Acute bronchiolitis, Acute gastroenteritis, Anxiety, Asthma, Attention deficit hyperactivity disorder, Autism, Croup, Depression, Diabetes, Eczema, Fever, Gastro-oesophageal reflux disease, Head injury, Otitis media, Tonsillitis, and Upper respiratory tract infection)*, with a further 400 shared between *Anxiety* (233 records) and *Depression* (167).

The proportion of medical records allocated to GPs varied by clinical condition, but GPs were routinely under-sampled, to ensure sufficient numbers of other healthcare settings. Any visit to a General Practitioner (GP) during the period 1 January 2012 to 31 December 2013 was considered. Without adjustment for the design effect, a minimum of 400 visits per condition was required to obtain national estimates with 95% Confidence Interval (CI) and precision of +/- 5%; as sampling for preventive care was opportunistic, no sample size could be pre-specified, but we anticipated that more than 400 of the 6400 records targeted for sampling would be eligible for assessment of preventive care indicators.

## 1.2 Sampling Process

A multistage stratified random sampling process was implemented. For logistical efficiency, sampling was conducted in three states, Queensland (QLD), New South Wales (NSW) and South Australia (SA), which together comprise 60.0% of the Australian population aged 15 years or younger in the 2012 and 2013 calendar years. State Departments of Health organize care within Health Districts: Hospital Health Services in QLD, Local Health Districts in NSW, and Local Health Networks in SA. For QLD, we targeted five Health Districts (two metropolitan, three regional), in NSW four Health Districts (two metropolitan, two regional), and in SA three Health Districts (two metropolitan, one regional).

## 1.3 Sampling of records

SA was over-sampled relative to population and allocated 100 records/condition (cf. 45 records expected on a population allocation), with NSW and QLD allocated the remaining 300 pro rata relative to population.^[2]^ Within each state, records were allocated to Metropolitan and Regional strata proportional to target populations, with the tertiary hospitals sharing the allocation appropriate to their geographical location.

The allocation of conditions to be sampled at each healthcare setting to achieve 400 per condition (or 233 anxiety and 167 for depression) was based on the following procedure:

1. Data on the number of episodes of care by condition and healthcare were sourced as follows: GP data were sourced through personal communications with an established survey of GP activity;^[3]^ data on SP occasions of care were estimated from published material;^[4]^ and ED and inpatient data were sourced from personal communications with the NSW Department of Health;^[5, 6]^
2. These data were reviewed by expert clinicians, and used to estimate the proportion of frequency of attendance by healthcare setting for each condition, as shown in Table 2^[[1]](#footnote-1)^ of the published protocol;^[2]^
3. HCP types with small numbers of attendances for a condition were over-sampled, and those with more numerous attendances were under-sampled, to create a revised sampling table that improved the accuracy of the point estimate for each healthcare setting.

The number of medical records targeted at GPs varied by condition, as the outcome of this process. Within the records selected at each GP, a single preventive care assessment was undertaken for each child that was eligible for one or more preventive care indicators. All visits in the medical record that was age-eligible for preventive care assessment was considered; routine ‘well-baby’ checks were, for example, therefore included in assessing preventive care.

Preventive care activities were expected at 2, 4, 6, 12, 18, 24 and 48 months of age. Each indicator was restricted to children of these ages. As shown in Table 2.1, different numbers of indicators are assessed at each time point.

Preventive care did not have a fixed date for an occasion of care, to allow for multi-episode assessments in a single record; surveyors were therefore directed to assign a dummy date of 31 December 2013 (i.e., the final date of the two-year survey period). Automated age-filtering of indicator questions, helped to reduce surveyors’ workload. As shown in eTable 1.1, assigning a dummy date and an eligibility window based on the dummy age effectively restricted sampling to specific birth date ranges; for example, assessments of actions planned at 2 months of age, with a dummy date of 31 December 2013, would only be offered to children born in the period 1 January 2013 to 31 October 2013. This procedure therefore artificially reduces the sample sizes of children eligible for assessment of preventive care, when compared to a method that assessed all children eligible for a preventive care assessment over the period 1 January 2012 to 31 December 2013.

**eTable 1.1: Number of indicators and follow-up duration for children of different ages**

| **Age-target for indicator action** | **Number of indicators** | **Child’s dummy age at indicator assessment** | **Eligibility window** | **Eligible birth dates** |
| --- | --- | --- | --- | --- |
| 2 months | 10 | 2-11 months | 10 months | 1 January 2013 –  31 October 2013 |
| 4 months | 10 | 4-11 months | 8 months | 1 January 2013 –  31 August 2013 |
| 6 months | 10 | 6-11 months | 6 months | 1 January 2013 –  30 June 2013 |
| 12 months | 4 | 12-23 months | 12 months | 1 January 2012 –  31 December 2012 |
| 18 months | 4 | 18-23 months | 6 months | 1 January 2012 –  30 June 2012 |
| 24 months | 4 | 24-35 months | 12 months | 1 January 2011 –  31 December 2011 |
| 48 months | 1 | 48-59 months | 12 months | 1 January 2009 –  31 December 2009 |

##

## 1.4 Sampling weights

There are two challenges in calculating an overall estimate of the appropriateness of preventive care. First, the different durations of eligibility windows for indicators at different age-points led to differential under-sampling of some age-points for preventive care (e.g., activities at 6 and 18 months were under-sampled by a factor of 4, while activities at 12, 24 and 48 months were only under-sampled by a factor of 2). Second, as sampling was performed *within* records selected because they were thought to contain one or more targeted clinical conditions, this imposes an age-structure on the sample, resulting in larger numbers eligible for some ages than for others; for example, there were ~100 children with one or more eligible GP records born in 2013, but around 300 in other years.

Sampling weights were constructed in such a way as to address both of these issues simultaneously. Assessments of 43 indicators were broken into 7 sub-groups: sub-groups at 2, 4 and 6 months of age each had ten indicators; sub-groups at 12, 18 and 24 months of age each had four indicators; and the sub-group at 48 months of age each contained one indicator.

Sampling fractions were calculated at Health District level, using identical methods, separately for each of the 7 sub-groups:

${Sampling Fraction}=Area Sampling Fraction \times Health District Sampling Fraction$

Where the Area Sampling Fraction (SF) refers to Metropolitan or Regional geographical areas, and:

$${Area SF}=\frac{\sum Population aged 0-15 years of selected Health Districts in an Area}{\text{Total population aged 0-15 years in an Area}}$$

There was only one Health District without any assessments for a targeted age: one of the two Health Districts in Regional NSW had no assessments at 6 months of age. The Area SF for the other Health District in Regional NSW was therefore adjusted by only including in the numerator the population for the Health District with an eligible record; the denominator was unchanged, as the whole population in the NSW Regional Area.

$${Health Districts SF}=\frac{\sum Preventive care sub-surveys in a Health District}{D}$$

The denominator (D) is the number of children aged 2, 4, 6, 12, 18, 24, or 48 months and expected to attend a GP in the period 1 January 2012 to 31 December 2013. This number was calculated as follows:

$D= Population \times Proportion attending GP at each targeted age$

Where:

***Population*** is the relevant denominator population in each Health District,^[7]^ aged 2, 4, 6, 12, 18, 24, or 48 months at any time during 1 January 2012 to 31 December 2013 – in each case this represents 24 months of births.

***Proportion attending GP*** was estimated from a secondary analysis of the Longitudinal Study of Australian Children, which linked MBS records to estimated probability of GP attendance.^[8]^ We estimated, from the published data, that children aged 2-18 months had a 68.3% probability of visiting a GP each year; in the absence of information on the proportion visiting GPs over two years, we assumed independence and estimated a 90.0% probability over two years. At 24-months of age the probability of GP visit in one year was 65.8% (88.3% over two years, assuming independence), and at 48-months of age 70.0% (91.0% over two years).

The Sampling Weight was calculated as the inverse of the Sampling Fraction. Multiplying sampling weights by the number of records assessed for preventive care at each age point gives the total number of children of that age expected to visit a GP in 2012 and 2013 in that Health District (or in the case of Regional NSW, that Area). In calculating the overall estimate of appropriateness, these weights therefore compensate for the differential sampling windows, and the fact that the underlying (17 condition) CTK sample has a different age-structure to that of all children visiting GPs who were eligible for the preventive care indicators assessed in this study.

## 1.5 Analysis

States was specified as strata, and the primary sampling unit (Health District) was specified as the clustering unit. Clustering by practice (in addition to clustering by GPs within a practice, and by clustering within a patient) occurs at a level below clustering by health district and is controlled for in the statistical analysis by adjusting for clustering at the primary sampling unit (i.e. the first level of clustering sampled, the health district). Adjusting for clustering is necessary to ensure that confidence intervals are not narrower than they should be. This approach is built into SAS and is referred to as the ‘ultimate cluster assumption’, providing an approximation to adjusting for clustering at multiple levels.^[9]^ Therefore, all sources of variability from subsequent stages of sampling are captured in the composite variance estimate under this assumption.^[10]^

Domain analysis was used for analysing both indicators and both types of bundles.^[10, 11]^ The overall estimate of appropriateness for preventive care was the weighted average of the indicator assessments.

## 1.6 Results

The table below summarises the sample sizes for each indicator question. This information is included in Table 2 of the manuscript, but is presented here in a summarised format focusing on the sample sizes by age of target assessment/intervention.

**eTable 1.2:** **Sample size for each indicator by target age for assessment/intervention.**

| Indicator Number | Age at which the assessment/intervention was targeted (months) | | | | | | |
| --- | --- | --- | --- | --- | --- | --- | --- |
|  | 2 | 4 | 6 | 12 | 18 | 24 | 48 |
| 1 | 98 |  |  |  |  |  |  |
| 2 |  | 91 |  |  |  |  |  |
| 3 |  |  | 80 |  |  |  |  |
| 4 | 99 |  |  |  |  |  |  |
| 5 |  | 91 |  |  |  |  |  |
| 6 |  |  | 80 |  |  |  |  |
| 7 | 99 |  |  |  |  |  |  |
| 8 |  | 91 |  |  |  |  |  |
| 9 |  |  | 79 |  |  |  |  |
| 10 | 99 |  |  |  |  |  |  |
| 11 |  | 91 |  |  |  |  |  |
| 12 |  |  | 80 |  |  |  |  |
| 13 | 99 |  |  |  |  |  |  |
| 14 |  | 91 |  |  |  |  |  |
| 15 |  |  | 80 |  |  |  |  |
| 16 | 99 |  |  |  |  |  |  |
| 17 |  | 91 |  |  |  |  |  |
| 18 |  |  | 80 |  |  |  |  |
| 19 | 99 |  |  |  |  |  |  |
| 20 |  | 91 |  |  |  |  |  |
| 21 |  |  | 80 |  |  |  |  |
| 22 | 99 |  |  |  |  |  |  |
| 23 |  | 91 |  |  |  |  |  |
| 24 |  |  | 79 |  |  |  |  |
| 25 | 99 |  |  |  |  |  |  |
| 26 |  | 91 |  |  |  |  |  |
| 27 |  |  | 80 |  |  |  |  |
| 28 |  |  |  | 279 |  |  |  |
| 29 |  |  |  |  | 140 |  |  |
| 30 |  |  |  | 279 |  |  |  |
| 31 |  |  |  |  | 140 |  |  |
| 32 |  |  |  | 279 |  |  |  |
| 33 |  |  |  |  | 141 |  |  |
| 34 |  |  |  | 279 |  |  |  |
| 35 |  |  |  |  | 141 |  |  |
| 36 |  |  |  |  |  | 289 |  |
| 37 |  |  |  |  |  | 290 |  |
| 38 |  |  |  |  |  | 290 |  |
| 39 | 101 |  |  |  |  |  |  |
| 40 |  | 92 |  |  |  |  |  |
| 41 |  |  | 83 |  |  |  |  |
| 42 |  |  |  |  |  | 313 |  |
| 43 |  |  |  |  |  |  | 273 |

## References:

1. Braithwaite J, Hibbert PD, Jaffe A, et al. Quality of health care for children in Australia, 2012-2013. *JAMA*. 2018;319(11):1113-24.

2. Hooper TD, Hibbert PD, Mealing N, Wiles LK, Jaffe A, White L, et al. CareTrack Kids-part 2. Assessing the appropriateness of the healthcare delivered to Australian children: study protocol for a retrospective medical record review. *BMJ Open*. 2015;5(4):e007749.

3. Britt H. Top 100 problems most frequently managed by General Practitioners, limited to patients 0-17, BEACH 2011-12 weighted data. [Personal communication] 2012.

4. Hiscock H, Roberts G, Efron D, Sewell JR, Bryson HE, Price AM, et al. Children Attending Paediatricians Study: a national prospective audit of outpatient practice from the Australian Paediatric Research Network. *Med J Aust*. 2011;194(8):392-7.

5. NSW Health. New South Wales Children’s and General Hospitals 2011–12. Inpatient admission data. [Personal communication]. Sydney: NSW Health; 2013.

6. NSW Health. New South Wales Children’s and General Hospitals 2011–12. SNOMED-CT Emergency department data. [Personal communication]. Sydney: NSW Health; 2013.

7. Queensland Health, New South Wales Health, South Australian Department of Health. Population estimates for children aged 0-15, by AU. [Personal communication] 2017.

8. Koyama L, Clifford S, Hiscock H, Carr S, Gold L, Wake M. The ecology of medical care for children in Australia. In: University of Melbourne, editor. University of Melbourne Scholarly Selective Student Monograph,. Melbourne, Australia; 2014.

9. Lewis TH. Complex Survey Data Analysis with SAS. Boca Raton, FL: CRC Press; 2016.

10. Heeringa SG, West BT, Berglund PA. Applied survey data analysis. Boca Raton: CRC Press; 2010.

11. Lohr S. Sampling: design and analysis. 2nd ed. Boston: Brooks/Cole; 2009.

1. The published protocol included occasions of care provided by clinical psychologists, for selected conditions; this aspect was deemed infeasible during implementation and removed. The nominal percentage of occasions of care allocated to psychologist was re-distributed, pro rata, to the other HCPs retained in the study. [↑](#footnote-ref-1)
