# Supplementary material for: The quality of preventive care for pre-school aged children in Australian general practice
Source: BMC Med. 2019 Dec 6;17:218. doi: 10.1186/s12916-019-1455-x (PMC6896286; doi:10.1186/s12916-019-1455-x)
Supplement: Supplementary file 2 — Additional file 2. Listing of indicator characteristics. eTable 2.1. Characteristics, by clinical indicator, 2012–2013. [file 12916_2019_1455_MOESM2_ESM.docx]

**Additional file 2: Listing of indicator characteristics**

**eTable 2.1:** Characteristics, by clinical indicator, 2012 - 2013

| **Indicator ID** | **Indicator Description** | **Age Inclusion Criteria** | **No. of GP sites** | **Strength of Recommendation^#^** | **Phase of Care** |
| --- | --- | --- | --- | --- | --- |
| PREV01 | Infants aged 2 months were weighed. | 2 - 11 months | 50 | Consensus-based recommendation | Screening |
| PREV02 | Infants aged 4 months were weighed. | 4 - 11 months | 48 | Consensus-based recommendation | Screening |
| PREV03 | Infants aged 6 months were weighed. | 6 - 11 months | 46 | Consensus-based recommendation | Screening |
| PREV04 | Infants aged 2 months had their length measured. | 2 - 11 months | 51 | Consensus-based recommendation | Screening |
| PREV05 | Infants aged 4 months had their length measured. | 4 - 11 months | 48 | Consensus-based recommendation | Screening |
| PREV06 | Infants aged 6 months had their length measured. | 6 - 11 months | 46 | Consensus-based recommendation | Screening |
| PREV07 | Infants aged 2 months had their head circumference measured. | 2 - 11 months | 51 | Consensus-based recommendation | Screening |
| PREV08 | Infants aged 4 months had their head circumference measured. | 4 - 11 months | 48 | Consensus-based recommendation | Screening |
| PREV09 | Infants aged 6 months had their head circumference measured. | 6 - 11 months | 46 | Consensus-based recommendation | Screening |
| PREV10 | Infants aged 2 months had their eyes examined. | 2 - 11 months | 51 | Consensus-based recommendation | Screening |
| PREV11 | Infants aged 4 months had their eyes examined. | 4 - 11 months | 48 | Consensus-based recommendation | Screening |
| PREV12 | Infants aged 6 months had their eyes examined. | 6 - 11 months | 46 | Consensus-based recommendation | Screening |
| PREV13 | Infants aged 2 months had their cardiovascular status examined. | 2 - 11 months | 51 | Consensus-based recommendation | Screening |
| PREV14 | Infants aged 4 months had their cardiovascular status examined. | 4 - 11 months | 48 | Consensus-based recommendation | Screening |
| PREV15 | Infants aged 6 months had their cardiovascular status examined. | 6 - 11 months | 46 | Consensus-based recommendation | Screening |
| PREV16 | Infants aged 2 months had their hips, limbs and joints examined. | 2 - 11 months | 51 | Consensus-based recommendation | Screening |
| PREV17 | Infants aged 4 months had their hips, limbs and joints examined. | 4 - 11 months | 48 | Consensus-based recommendation | Screening |
| PREV18 | Infants aged 6 months had their hips, limbs and joints examined. | 6 - 11 months | 46 | Consensus-based recommendation | Screening |
| PREV19 | Infants aged 2 months had their developmental progress examined. | 2 - 11 months | 51 | Consensus-based recommendation | Screening |
| PREV20 | Infants aged 4 months had their developmental progress examined. | 4 - 11 months | 48 | Consensus-based recommendation | Screening |
| PREV21 | Infants aged 6 months had their developmental progress examined. | 6 - 11 months | 46 | Consensus-based recommendation | Screening |
| PREV22 | Infants aged 2 months had any parental concerns documented. | 2 - 11 months | 51 | Consensus-based recommendation | Screening |
| PREV23 | Infants aged 4 months had any parental concerns documented. | 4 - 11 months | 48 | Consensus-based recommendation | Screening |
| PREV24 | Infants aged 6 months had any parental concerns documented. | 6 - 11 months | 45 | Consensus-based recommendation | Screening |
| PREV25 | Infants aged 2 months had their nutrition assessed. | 2 - 11 months | 51 | Grade B | Screening |
| PREV26 | Infants aged 4 months had their nutrition assessed. | 4 - 11 months | 48 | Grade B | Screening |
| PREV27 | Infants aged 6 months had their nutrition assessed. | 6 - 11 months | 46 | Grade B | Screening |
| PREV28 | Infants aged 12 months were weighed. | 1 year | 70 | Consensus-based recommendation | Screening |
| PREV29 | Infants aged 18 months were weighed. | 18 - 23 months | 59 | Consensus-based recommendation | Screening |
| PREV30 | Infants aged 12 months had their height measured. | 1 year | 70 | Consensus-based recommendation | Screening |
| PREV31 | Infants aged 18 months had their height measured. | 18 - 23 months | 59 | Consensus-based recommendation | Screening |
| PREV32 | Infants aged 12 months had their eyes and vision examined. | 1 year | 70 | Consensus-based recommendation | Screening |
| PREV33 | Infants aged 18 months had their eyes and vision examined. | 18 - 23 months | 59 | Consensus-based recommendation | Screening |
| PREV34 | Infants aged 12 months had their developmental progress examined. | 1 year | 70 | Consensus-based recommendation | Screening |
| PREV35 | Infants aged 18 months had their developmental progress examined. | 18 - 23 months | 59 | Consensus-based recommendation | Screening |
| PREV36 | Children aged 2 years were weighed. | 2 years | 69 | Grade C | Screening |
| PREV37 | Children aged 2 years had their height measured. | 2 years | 70 | Grade C | Screening |
| PREV38 | Children aged 2 years had their development and behaviour assessed. | 2 years | 70 | Grade C | Screening |
| PREV39 | Infants aged 2 months received immunisation according to Australian DOHA immunisation schedule. | 2 - 11 months | 51 | Consensus-based recommendation | Treatment |
| PREV40 | Infants aged 4 months received immunisation according to Australian DOHA immunisation schedule. | 4 - 11 months | 49 | Consensus-based recommendation | Treatment |
| PREV41 | Infants aged 6 months received immunisation according to Australian DOHA immunisation schedule. | 6 - 11 months | 48 | Consensus-based recommendation | Treatment |
| PREV42 | Children aged 2 years received immunisation according to Australian DOHA immunisation schedule. | 2 years | 76 | Consensus-based recommendation | Treatment |
| PREV43 | Children aged 4 years are immunised according to Australian DOHA immunisation schedule. | 4 years | 75 | Consensus-based recommendation | Treatment |

Legend: ID=Identifier; GP=General Practitioner; DOHA=Australian Government Department of Health and Ageing.

# Strength of recommendation as reported in individual CPGs. CPGs used a variety of classification schemes for allocating strength of recommendation in Grades (with A indicating the strongest recommendation in all classification schemes). Where Strength of Recommendation, or Level of Evidence, were not specified in the CPG, the term “Consensus-based recommendation” was assigned.
