# Supplementary material for: The quality of preventive care for pre-school aged children in Australian general practice
Source: BMC Med. 2019 Dec 6;17:218. doi: 10.1186/s12916-019-1455-x (PMC6896286; doi:10.1186/s12916-019-1455-x)
Supplement: Supplementary file 3 — Additional file 3. Definitions for the criteria against which reviewers marked indicator compliance. [file 12916_2019_1455_MOESM3_ESM.docx]

**Additional file 3: Definitions for the criteria against which reviewers marked indicator compliance**

**Department of Health and Ageing National Immunisation Schedule (as at May 2012)**

| Birth | - Hepatitis B (hepB) |
| --- | --- |
| 2 months | - Hepatitis B (hepB) - Diphtheria, tetanus and whooping cough (acellular pertussis) (DTPa) - *Haemophilus influenzae* type b (Hib) - Polio (inactivated poliomyelitis) (IPV) - Pneumococcal conjugate (13VPCV) - Rotavirus |
| 4 months | - Hepatitis B (hepB) - Diphtheria, tetanus and whooping cough (acellular pertussis) (DTPa) - *Haemophilus influenzae* type b (Hib) - Polio (inactivated poliomyelitis) (IPV) - Pneumococcal conjugate (13VPCV) - Rotavirus |
| 6 months | - Hepatitis B (hepB) - Diphtheria, tetanus and whooping cough (acellular pertussis) (DTPa) - *Haemophilus influenzae* type b (Hib) - Polio (inactivated poliomyelitis) (IPV) - Pneumococcal conjugate (13VPCV) - Rotavirus |
| 12 months | - *Haemophilus influenzae* type b (Hib) - Measles, mumps and rubella (MMR) - Meningococcal C (MenCCV) |
| 18 months | - Chickenpox (varicella) (VZV) |
| 4 years | - Diphtheria, tetanus and whooping cough (acellular pertussis) (DTPa) - Polio (inactivated poliomyelitis) (IPV) - Measles, mumps and rubella (MMR) |

**Eye examination:**

*2, 4 and 6 months -* observation, fixation and following

*12 and 18 months -* observation, fixation and following, corneal light reflex

**Nutrition assessment:** breastfeeding and solids

**Oral health examination:** teeth and gums

**Parental concerns:** “any parental concerns” can relate to specific or general signs, symptoms and presentations (e.g. a new condition/illness, or general developmental progress).

**Other care providers:**

If it is noted within GP records that components of preventive care have been delivered by other primary care clinicians external to the practice (i.e. not including their own practice nurses), IQs for these care components should be assessed as ‘not applicable’.

**Care refusal:**

If the GP offered recommended care that was refused by the parent or child, the relevant IQ(s) should be recorded as compliant (i.e. Yes).
